# Миокины и адипомиокины: медиаторы воспаления или уникальные молекулы таргетной терапии ожирения?

**DOI:** 10.14341/probl12779

**Published:** 2021-08-10

**Authors:** О. В. Васюкова, Ю. В. Касьянова, П. Л. Окороков, О. Б. Безлепкина

**Affiliations:** Национальный медицинский исследовательский центр эндокринологии; Национальный медицинский исследовательский центр эндокринологии; Национальный медицинский исследовательский центр эндокринологии; Национальный медицинский исследовательский центр эндокринологии

**Keywords:** миокины, физические нагрузки, миостатин, ирисин, ИЛ-6, декорин, FGF-21, ожирение

## Abstract

Скелетная мускулатура составляет около 25% общей массы у детей и более 40% — у взрослых. Исследования последних 20 лет показали, что наряду с основными функциями мышечная ткань обладает гормональной активностью. Установлено, что миоциты способны высвобождать сигнальные молекулы — миокины. Они действуют ауто- и паракринно в пределах мышцы, а при высоком уровне — через системную циркуляцию, осуществляя взаимодействия между скелетными мышцами и различными органами и тканями, такими как печень, костная и жировая ткань, головной мозг. Доказано, что ключевым фактором экспрессии миокинов является физическая нагрузка, а их уровень во многом зависит от физической тренированности, количества скелетной мышечной массы и ее состава (соотношение быстрых и медленных волокон), от интенсивности и продолжительности физических нагрузок. Миокины имеют широкий спектр физиологических эффектов: миостатин подавляет рост и дифференцировку мышечной ткани, а декорин, действуя как его антагонист, способствует гипертрофии мышц. Интерлейкин-6 обеспечивает энергетическим субстратом сокращающиеся мышечные волокна, фактор роста фибробластов 21 активирует механизмы получения энергии при голодании и улучшает чувствительность тканей к инсулину; ирисин стимулирует термогенез, поглощение глюкозы миоцитами, а также способствует повышению минеральной плотности костной ткани. Изучение миокинов является одним из ключевых звеньев в понимании механизмов, лежащих в основе ожирения и метаболических осложнений, последствий малоподвижного образа жизни, а также реализации действия физической активности. Учитывая физиологические эффекты миокинов в организме, в перспективе они могут стать мишенями для терапии данных состояний.

## Abstract (en)

Skeletal muscles make up about 25% of the total mass in children and more than 40% in adults. Studies of the last twenty years have shown that along with the main functions, muscle tissue has hormonal activity. It was found that myocytes are able to release signaling molecules-myokines. They act auto-and paracrine within the muscle, and at a high level-through the systemic circulation, carrying out interactions between skeletal muscles and various organs and tissues, such as the liver, bone and adipose tissue, the brain. It is proved that the key factor in the expression of myokines is physical activity, and their level largely depends on physical fitness, the amount of skeletal muscle mass and its composition (the ratio of fast and slow fibers), on the intensity and duration of physical activity. Myokines have a wide range of physiological effects: myostatin suppresses the growth and differentiation of muscle tissue, and decorin, acting as its antagonist, promotes muscle hypertrophy. Interleukin 6 provides an energy substrate for contracting muscle fibers, fibroblast growth factor 21 activates the mechanisms of energy production during fasting and improves tissue sensitivity to insulin; irisin stimulates thermogenesis, glucose uptake by myocytes, and also contributes to an increase in bone mineral density. The study of myokines is one of the key links in understanding the mechanisms underlying obesity and metabolic complications, the consequences of a sedentary lifestyle, as well as the implementation of the action of physical activity. Taking into account the physiological effects of myokines in the body, in the future they can become therapeutic targets for the treatment of these conditions.

Миокины были открыты в начале 2000-х гг. и представляют собой белки, синтезируемые скелетными мышцами при их сокращении [1]. Обладая аутокринными и паракринными эффектами, а также возможностью при определенных концентрациях оказывать системное действие через собственные рецепторы в мышцах, жировой ткани, печени, поджелудочной железе, сердце, костной ткани, иммунных клетках и клетках головного мозга, миокины обеспечивают метаболическое взаимодействие между данными органами, тканями и имеют широкий спектр физиологических эффектов.

В настоящее время известно более 1000 миокинов, относящихся к различным структурно-функциональным группам (цитокины, хемокины, семейство простагландинов и др.).

Часть миокинов, помимо клеток скелетной мышечной ткани, синтезируется и секретируется адипоцитами, в связи с чем выделяют миокины и адипомиокины. Соответственно, последние могут оказывать как отрицательное метаболическое воздействие, выступая в роли провоспалительных адипокинов при ожирении, так и положительное, повышаясь в ответ на физические упражнения.

Известно, что ожирение в сочетании с низким уровнем физической активности приводит к избыточному накоплению висцеральной жировой ткани и развитию метаболических осложнений. Патогенетической основой данных изменений является развитие системного воспаления, характеризующегося клеточной инфильтрацией, фиброзом, изменениями микроциркуляции, сдвигом секреции адипокинов и метаболизма в жировой ткани, а также накоплением в крови таких неспецифических маркеров воспаления, как С-реактивный белок, фибриноген, лейкоциты, уровень которых отражает выраженность процесса [2][3], приводящего к развитию инсулинорезистентности в периферических тканях [4].

Однако повышающиеся в ответ на физическую нагрузку миокины могут уравновешивать провоспалительные эффекты адипокинов. При сокращении мышечные волокна экспрессируют ирисин, интерлейкин-6, фактор роста фибробластов 21 и др., которые оказывают свое воздействие не только локально в мышцах, но и в органах-мишенях, уменьшая развитие воспаления.

Миокины опосредуют связь между мышцами и печенью, жировой тканью, поджелудочной железой, головным мозгом, другими органами. Современные исследования демонстрируют активное участие миокинов в регуляции процессов липолиза, глюконеогенеза, секреции инсулина бета-клетками поджелудочной железы, активации термогенеза [5–7].

Известно, что малоподвижный образ жизни связан с развитием ожирения, сахарного диабета 2 типа, сердечно-сосудистых заболеваний, остеопороза и ранней смертностью, а регулярные физические нагрузки способствуют профилактике данных состояний [8].

В настоящее время активно изучаются молекулярные механизмы этих взаимодействий, и предполагается, что именно миокины являются основным патогенетическим звеном, обеспечивающим положительное влияние физических упражнений на здоровье.

Адаптируясь к механическим, нервным и гуморальным воздействиям, скелетная мускулатура играет решающую роль в обеспечении физической активности, расходовании энергии и утилизации глюкозы [9]. Физические упражнения и анаболические гормоны, такие как инсулин, инсулиноподобный фактор роста 1, гормон роста и тестостерон, увеличивают массу скелетных мышц. И наоборот, гиподинамия, развивающаяся вследствие нервно-мышечных заболеваний, старения, хронических заболеваний (почечная недостаточность, дыхательная недостаточность, тяжелый сахарный диабет, гиперкортицизм и др.) приводит к дефициту и атрофии мышц (рис. 1).

**Figure:**
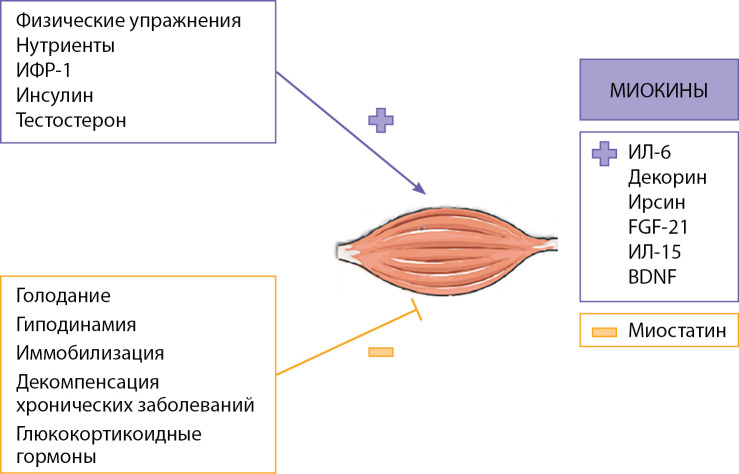
Рисунок 1. Факторы, оказывающие положительное (+) и отрицательное (-) действие на состояние мышечной ткани.ИФР-1 — инсулиноподобный фактор роста 1; ИЛ — интерлейкин; BDNF — мозговой нейротрофический фактор; FGF-21 — фактор роста фибробластов 21.

Таким образом, мышечная ткань вовлечена в тесное взаимодействие с различными органами и системами, что, с одной стороны, дает понимание патогенетических основ положительного влияния физической нагрузки на организм в целом и профилактику различных ассоциированных с гиподинамией заболеваний, а с другой — возможность узнать молекулярные механизмы развития метаболических осложнений.

Но вместе с тем до настоящего времени остается неясным, как и почему уровни провоспалительных адипокинов, с одной стороны, повышаются в состоянии ожирения, а с другой — оказывают благотворное воздействие на организм после физической нагрузки.

Кроме этого, список предполагаемых миокинов продолжает расти, однако специфические физиологические и патологические эффекты этих молекул у человека малоизучены. Остаются вопросы, является ли скелетная мышца основным или единственным источником данного миокина, как регулируется его локальная и системная концентрация, существуют ли биологические различия между видами и какие конкретные сигнальные механизмы опосредуют биологические эффекты миокина в различных органах.

И в то же время несомненно, что лучшее понимание действия миокинов может определить новые методы лечения ожирения, сахарного диабета, сердечно-сосудистых и других заболеваний.

В данном обзоре представлены основные эффекты известных на сегодняшний день миокинов, а также рассмотрены их изменения при разных состояниях у людей.

## МИОСТАТИН

Миостатин является первым миокином, открытым в 1997 г. и полученным из мышечной ткани [10].

Миостатин, или фактор роста и дифференцировки 8 (myostatin, growth differentiation factor 8, GDF8), является членом суперсемейства TGF-beta/BMP (трансформирующий фактор роста бета костного морфогенетического белка), взаимодействует с рецепторами ACVR2B (activin type II receptor, активиновый рецептор II типа) и связывается фоллистатин-подобным белком-3 (FSTL3).

Миостатин приводит к снижению роста мышечной ткани путем подавления пролиферации, дифференцировки миоцитов и синтеза белка [11][12], а также оказывает системное воздействие на организм.

Механизм реализации данных эффектов миостатина связан с активацией факторов транскрипции семейства Smad (Smad2 и Smad3), Forkhead Box — FOXO (1, 2 и 3) и ингибированием пути AKT/mTOR [13]. После физических нагрузок отмечается повышение PGC-1α (Peroxisome proliferator-activated receptor gamma coactivator 1-alpha), который стимулирует митохондриальный биогенез, связывается с FOXO и ингибирует его транскрипционную активность [14], тем самым препятствуя распаду мышечных белков.

Инактивирующая мутация гена миостатина (MSTN) приводит к двукратному увеличению всех скелетных мышц (в виде гипертрофии и гиперплазии мышечных волокон). Данная мутация была описана у крупного рогатого скота, овец, собак и человека [15–17]. Напротив, гиперэкспрессия гена миостатина (MSTN) у трансгенных мышей приводит к снижению мышечной массы.

Также стоит отметить, что при физической нагрузке уровень миостатина снижается, тем самым стимулируя процесс роста мышечной ткани. Его концентрация в миоцитах мышей уменьшается после беговой нагрузки, способствуя росту и дифференцировке сателлитных клеток [18–20]. Подобные результаты были получены и у людей [21][22].

Эффекты миостатина не ограничиваются скелетными мышцами. Известно, что мРНК миостатина экспрессируется в жировой ткани, хотя уровень его существенно ниже, чем в скелетной мускулатуре [10]. В исследованиях in vitro показан разный профиль экспрессии компонентов сигнального пути миостатина (ACVR2B, FSTL3) в висцеральной и подкожной жировой клетчатке у мышей [23].

Также отмечена его роль в регуляции роста адипоцитов. Поскольку мышечная и жировая ткань развиваются из одних и тех же мезенхимальных стволовых клеток, в экспериментах in vitro миостатин ингибирует миогенез и стимулирует адипогенез данных клеток, а при действии на преадипоциты, наоборот, препятствует их дифференцировке [24].

Tingqing Guo и соавт. (2009г) исследовали влияние ингибирования передачи сигналов миостатина в скелетных мышцах и в жировой ткани на композиционный состав тела, метаболический профиль [25]. Так, у мышей с делецией гена миостатина (Mstn-/-) выявлены увеличение мышечной массы и снижение жировой, улучшение показателей углеводного и липидного обмена на нормо- и высококалорийной диете, а также устойчивость к набору веса и развитию инсулинорезистентности, что не наблюдалась при блокировании передачи сигнала миостатина в жировой ткани. У Mstn-/- мышей отмечались более низкий уровень глюкозы и инсулина натощак, более высокая скорость инфузии глюкозы во время клэмп-теста. Полученные результаты свидетельствуют об улучшении чувствительности тканей к инсулину, что ведет к увеличению поглощения глюкозы мышечной и жировой тканями, связанному с повышением фосфорилирования серин/треониновой протеинкиназы В (Akt). Позднее (2016 г.) авторы опубликовали результаты исследования композиционного состава тела и обмена веществ. У мышей с дефицитом миостатина были выявлены нормальная скорость основного обмена и более высокий дыхательный коэффициент, что свидетельствует о повышенной скорости окисления углеводов; также отмечено увеличение количества тощей массы и низкое содержание жировой массы за 15 мес наблюдений [26]. В другой работе у мышей с дефицитом миостатина было отмечено повышение чувствительности тканей к инсулину благодаря увеличению активности AMPK в мышцах [27].

В костной ткани миостатин приводит к резорбции, усиливая остеокластогенез и препятствуя остеобластогенезу. Так, у мышей с инактивирующими мутациями гена миостатина отмечалось увеличение плотности костной ткани. Предположительный механизм данного эффекта исследован в работе Y. Qin и соавт. Было показано, что миостатин подавляет экспрессию микроРНК-218 в экзосомах остеоцитов, увеличивает выработку склеростина, RANKL (лиганд рецептора-активатора ядерного фактора каппа-В) и Dickkopf-связанного белка-1 (DKK1), ингибируя сигнальный путь Wnt/β-катенин и ускоряя RANKL-опосредованное образование остеокластов [28].

Таким образом, миостатин оказывает отрицательное действие на рост мышечной ткани и формирование костной массы, углеводный обмен, способствует адипогенезу.

Современные исследования направлены на разработку препаратов, блокирующих сигнальные пути миостатина, и изучение возможностей их применения в терапии нервно-мышечных заболеваний, ожирения, ортопедической патологии, при снижении мышечной массы и мышечной силы.

## ДЕКОРИН

Декорин — это белок с молекулярной массой 90–140 кДа, относящийся к семейству богатых лейцином протеогликанов, связанный с фибриллами коллагена во всех соединительных тканях. Ген, кодирующий декорин (DCN), регулирует активность трансформирующего фактора роста бета 1 (TGF -beta 1), а также клеточный цикл [29].

Декорин секретируется миоцитами и высвобождается в кровь в ответ на сокращение мышечных волокон. Он действует как антагонист миостатина, стимулирует пролиферацию и дифференцировку миобластов [19].

У людей экспрессия мРНК декорина и его уровень в сыворотке повышаются как после однократной физической нагрузки, так и после регулярных тренировок [30].

Известно также, что декорин увеличивает экспрессию фоллистатина — еще одного регулятора роста скелетных мышц. Фоллистатин непосредственно связывает миостатин, блокируя его ингибирующее действие на рост мышечной ткани [31]. В исследованиях in vitro показано, что декорин совместно с фоллистатином уменьшают развитие фиброза скелетных мышц и способствуют дифференцировке мышечных волокон [32].

## ФАКТОР РОСТА ФИБРОБЛАСТОВ ЧЕЛОВЕКА 21 (FGF-21)

FGF-21 является членом суперсемейства факторов роста фибробластов — белков, участвующих в пролиферации, росте и дифференцировке клеток.

Первоначально считалось, что он секретируется исключительно клетками печени. В дальнейшем был показан широкий диапазон экспрессии мРНК FGF-21 адипоцитами, мышечной тканью, поджелудочной железой, в головном мозге.

Для осуществления эффектов FGF-21 требуются два компонента: одна из изоформ рецептора FGF (FGFR1c и FGFR3c) и кофактор бета-Klotho (KLB), совместно активирующие последующие сигнальные пути. У людей FGFR1c и FGFR3c экспрессируются повсеместно, тогда как экспрессия KLB ограничена печенью, жировой и костной тканью, головным мозгом, но отсутствует в мышцах [33].

В исследованиях была отмечена роль FGF-21 в активации кетогенеза, глюконеогенеза и β-окисления липидов при голодании [34–36]. У человека выявлено повышение уровня FGF-21 после 7 дней без приема пищи [37]. Повышение уровня свободных жирных кислот во время голодания активирует PPAR-α (Peroxisome proliferator-activated receptor-α), что стимулирует синтез и секрецию FGF-21.

В последние годы исследования показали, что FGF-21 участвует в регуляции углеводного и липидного обмена, рассматривается перспективной терапевтической мишенью для лечения ожирения и метаболических осложнений [38], в том числе неалкогольной жировой болезни печени (НАЖБП) [39][40].

В работе Kharitonenkov A. и соавт. FGF-21 в белой жировой ткани увеличивал экспрессию GLUT-4 и поглощение глюкозы, а у мышей c гипергликемией и инсулинорезистентностью ob/ob и db/db (мыши дикого типа и мыши с дефицитом лептина) инъекция FGF-21 снижала уровень глюкозы и триглицеридов в течение 24 ч [33].

При проведении клэмп-теста у здоровых людей, пациентов с нарушением толерантности к глюкозе (НТГ) и сахарным диабетом (СД) 2 типа отмечалось повышение уровня FGF-21 в сыворотке и его мРНК в скелетных мышцах, что свидетельствует о стимулирующем влиянии инсулина на секрецию FGF-21. При этом более высокие концентрации FGF-21 имели лица с нарушением углеводного обмена. Так, у пациентов с СД 2 типа и НТГ повышенный уровень FGF-21 положительно коррелировал с глюкозой, инсулином натощак, индексом НОМА, триглицеридами (ТГ) и отрицательно — с уровнем липопротеидов высокой плотности (ЛПВП) [41].

Вместе с тем у лиц с СД 2 типа отмечается сниженный уровень биологически активного FGF-21 по отношению к общему количеству FGF-21 в ответ на пероральный глюкозотолерантный тест (ПГТТ), что связывают с повышенным уровнем белка, активирующего фибробласты (fibroblast activation protein α, FAP) в сыворотке у данных пациентов [42].

Физические упражнения оказывают стимулирующее влияние на экспрессию FGF-21 и повышают его уровень в сыворотке [5]. Метаанализ 2020 г., включивший семь исследований с участием 125 взрослых пациентов (21–64 года) с нормальной, избыточной массой тела и ожирением, показал, что однократные физические нагрузки (ФН) увеличивают уровень FGF-21 в сыворотке независимо от массы тела. При этом повышенный уровень FGF-21 сохраняется в течение 1 ч и снижается до уровня, близкого к исходным значениям, через 3 ч [43]. Однако у пациентов с СД 2 типа не отмечалось повышения уровня FGF-21 [44].

Таким образом, особенностями экспрессии FGF-21 у лиц с ожирением и нарушением углеводного обмена являются повышенный базальный уровень данного белка, связанный с наличием инсулинорезистентности, а также сниженный ответ FGF-21 после ФН. На сегодняшний день показана роль FGF-21 в активации механизмов получения энергии при голодании, положительном влиянии на углеводный и липидный обмен, в реализации позитивных эффектов физических нагрузок у здоровых людей и лиц с ожирением без нарушений углеводного обмена.

## ИРИСИН

Ирисин — миокин-адипокин, открытый в 2012 г. группой исследователей Böstrom Р. и соавт. [45]. Он представляет собой полипептид из 112 аминокислот, который отщепляется от FNDC5 (белка 5, содержащего домен фибронектина III типа) путем протеолиза при стимуляции PGC1-α (коактиватор PPARγ — рецептор, активирующий пролиферацию пероксисом), а затем секретируется в кровоток. Следует отметить, что протеолитический фермент в настоящее время остается неизвестным. PGC1-α через активацию PPAR-γ повышает экспрессию разобщающего белка 1 — термогенина (UCP1), что приводит к повышению несократительного термогенеза и расхода энергии. Поэтому первоначально ирисин был заявлен главным белком «браунинга» («browning») — превращения белой жировой ткани (БЖТ) в бурую (БурЖТ) и бежевую (БежЖТ), которые отличаются большим количеством митохондрий, высокой скоростью окислительных процессов и являются наиболее активными в процессах термогенеза и рассеивания тепла, что в эксперименте приводило к снижению массы тела и повышению чувствительности тканей к инсулину. Также в исследованиях in vitro и in vivo у животных ирисин повышал экспрессию генов, отвечающих за морфологические особенности и митохондриальную активность БурЖТ [45].

Однако полученные положительные результаты на мышах в отношении «браунинга» в настоящее время не доказаны у людей [46]. Данный факт связывают с несколькими причинами. Так, адипокины, происходящие от разных клеток-предшественников, имеют различный паттерн экспрессии генов, отвечающих за термогенез [47]. Адипоциты, в зависимости от их топографии, по-разному экспрессируют рецептор интегрин αV/β5, участвующий в передаче сигнала ирисина. Помимо этого, эпигенетические факторы, адипокины жировой ткани могут влиять на дифференцировку адипоцитов и сигнальные пути ирисина [49]. В связи с этими и другими причинами использование ирисина в качестве терапии ожирения остается предметом дальнейших исследований, как и сама возможность «браунинга» у людей.

Основными источниками ирисина у человека являются скелетная мышечная ткань (СМТ) и белая жировая ткань [50]. У человека высокая экспрессия FNDC5 отмечается в СМТ, а также в других органах, содержащих мышечную ткань (сердце, язык, прямая кишка), более низкая — в печени и поджелудочной железе [51]. При этом экспрессия гена FNDC5 в миоцитах в 200 раз выше, чем в адипоцитах [50].

Помимо «браунинга», описаны многочисленные положительные метаболические эффекты ирисина у животных. В СМТ он стимулирует поглощение глюкозы миоцитами и окисление свободных жирных кислот (СЖК), обеспечивая необходимым энергетическим субстратом работающие мышцы, а в печени ингибирует глюконеогенез и стимулирует гликогенолиз [52]. Механизм утилизации глюкозы миоцитами связан со снижением внутриклеточного уровня АТФ, последующим фосфорилированием АМПК (5’АМФ-активируемой протеинкиназы), активирующей MAPK (митоген-активируемую протеинкиназу p38), которая стимулирует процесс транслокации GLUT-4 в мембраны клеток [53].

Исследования на животных показали, что ирисин повышает толерантность к глюкозе и снижает инсулинорезистентность (ИР) [54]. Также ирисин стимулирует липолиз с помощью гормончувствительной липазы (HSL, hormone sensitive lipase) и ингибирует липогенез в адипоцитах мышей [55], что способствует снижению количества жировой ткани.

В работе Miyamoto-Mikami E. и соавт. у здоровых взрослых после 8 нед тренировок на выносливость повышение уровня циркулирующего ирисина положительно коррелировало со снижением жировой массы [56].

Ирисин также оказывает противовоспалительное действие в адипоцитах и макрофагах, повышает их способность к фагоцитозу, подавляет экспрессию провоспалительных цитокинов, что также способствует снижению количества жировой ткани. Более того, антиоксидантные и противовоспалительные эффекты ирисин оказывает на гепатоциты, что могло бы быть полезно в снижении активности стеатогепатита [57].

Отдельного внимания заслуживают особенности секреции ирисина у пациентов с ожирением и СД 2 типа. В большинстве исследований сообщается, что при избытке массы тела уровень ирисина положительно коррелирует с индексом массы тела (ИМТ) [7][51][52][58–61]. Так, более высокие концентрации ирисина в сыворотке отмечаются у людей с ожирением, а пациенты с нервной анорексией имеют на 15% более низкие уровни ирисина в сыворотке по сравнению с нормальным весом и на 30% — по сравнению с морбидным ожирением. Кроме того, ирисин положительно коррелирует с количеством жировой ткани, окружностью талии, соотношением талии и бедер [7][58][61][62], мышечной массой [7][51], а также с глюкозой натощак и индексами инсулинорезистентности [51][63–65], при этом индекс НОМА и количество безжировой ткани являются основными предикторами высокого уровня ирисина. Это вполне объяснимо, так как при ожирении наряду с повышением массы жировой ткани увеличивается и «тощая» (безжировая) масса. Однако существует и другая точка зрения [66]. Предполагается, что при ожирении основным источником ирисина становятся адипоциты, и увеличение жировой массы стимулирует его продукцию, чтобы противодействовать нарушению энергетического баланса при избытке массы тела. Кроме этого, повышение уровня ирисина может быть компенсаторным механизмом в ответ на развитие резистентности к нему и способствует повышению чувствительности тканей к инсулину [66].

Предполагается, что ирисин играет важную роль в поддержании функции β-клеток поджелудочной железы. Он повышает экспрессию В-трофина — гормона, способствующего пролиферации и снижению апоптоза β-клеток поджелудочной железы [54]. При развитии СД 2 типа данный механизм нарушается.

Разные профили ирисина в сыворотке отмечаются у пациентов с СД 1 и 2 типов. При СД 1 типа у пациентов с нормальной массой тела уровень ирисина выше, чем в контроле [67][68]. А при СД 2 типа отмечаются более низкие уровни ирисина по сравнению с контрольной группой [64][69–71]. Также более низкие уровни ирисина отмечаются у пациентов с предиабетом [72]. Кроме того, низкий уровень ирисина ассоциирован с микрососудистыми осложнениями: диабетической нефропатией, ретинопатией.

Таким образом, у пациентов с ожирением отмечается компенсаторное повышение уровня ирисина в сыворотке, а при развитии СД 2 типа, несмотря на сохраняющееся ожирение, отмечаются низкие уровни данного белка. Это может быть связано со снижением экспрессии PGC-1a, который воздействует на FNDC5 и синтез ирисина в скелетных мышцах у данных пациентов [73]. Кроме этого, у пациентов с СД 2 типа значительно снижены экспрессия гена FNDC5 в мышцах и уровень мРНК FNDC5 [50].

Поскольку одним из ключевых факторов, влияющих на экспрессию PGC1-α, усиливающего термогенез за счет повышения UCP-1, является физическая нагрузка (ФН), во многих исследованиях изучалось ее влияние на секрецию ирисина.

При исследовании у животных отмечалось выраженное повышение уровня ирисина в сыворотке и уменьшение количества жировой массы после ФН [74][75] . Кроме того, регулярные физические упражнения значимо повышали уровни экспрессии PGC-1-α и FNDC5 в скелетных мышцах животных при нормокалорийном питании и диете с повышенным содержанием жиров по сравнению с контролем [76].

В большинстве исследований у людей отмечено повышение уровня ирисина в сыворотке после однократных аэробных и силовых упражнений. В работе Huh J.Y. и соавт., включившей 117 здоровых взрослых женщин, отмечено повышение уровня сывороточного ирисина через 30 минут после однократных интенсивных аэробных упражнений в ответ на снижение уровня аденозинтрифосфата в мышцах, тогда как после регулярных физических нагрузок (в течение 8 нед) его уровень значимо не повышался [51]. В работе Löffler D. и совт. у подростков с ожирением уровень ирисина в сыворотке увеличивался на 60% после 45-минутной аэробной тренировки, но не менялся значимо при регулярных тренировках через 6 нед; однако через год ФН отмечено его повышение [7]. В работе Bluher S. и соавт., включившей 65 детей 7–18 лет (54% мальчики) с ожирением, отмечалось повышение концентрации ирисина (на 12% [6][17], р=0,00003) при снижении веса после одного года регулярных ФН и сбалансированного питания, однако корреляции между ирисином и SDS ИМТ, адипокинами, маркерами воспаления не отмечалось [77]. Учитывая, что главным предиктором уровня ирисина считается количество мышечной ткани, ее увеличение на фоне регулярных ФН может объяснять полученные результаты.

Помимо ФН, на уровень FNDC5 и ирисина также влияет изменение уровня лептина. В работе Rodríguez A. и соавт. инъекции лептина у мышей вызывали повышение экспрессии FNDC5 скелетных мышц и уровня ирисина, тем самым стимулируя миогенез (повышая экспрессию генов мионектина и миогенина, снижая мРНК миостатина) и увеличение количества мышечной массы, при этом было отмечено снижение экспрессии FNDC5 в подкожножировой клетчатке, а также стимулированной ирисином экспрессии генов БурЖТ (Ucp1 и Cidec) и БежЖТ (Tmem26), что препятствует процессу «браунинга» [78]. У людей уровень ирисина положительно коррелирует с уровнем лептина и отрицательно — с адипонектином как у лиц с ожирением, так и с нормальным весом [79].

Интересно отметить, что концентрация ирисина в сыворотке не изменяется в течение суток и после приема пищи. При этом его уровень уменьшается с возрастом и имеет гендерные различия: у мужчин он выше, чем у женщин, что также можно объяснить физиологическими особенностями композиционного состава тела [7].

Ирисин также положительно влияет на костную ткань как у людей, так и у животных [80][81]. Было показано повышение минеральной плотности костной ткани (МПК) за счет активации костных остеобластов и снижения ингибиторов остеобластогенеза [82][83].

Таким образом, ирисин имеет широкий спектр физиологических эффектов на организм. Он обеспечивает энергетическим субстратом сокращающиеся скелетные мышцы, участвует в процессе миогенеза, оказывает противовоспалительное действие, повышает МПК и расход энергии, улучшает углеводный обмен, в связи с чем в настоящее время остается предметом многочисленных исследований.

## ИНТЕРЛЕЙКИН-6 (ИЛ-6)

ИЛ-6 относится к подсемейству цитокинов, включающему также ИЛ-11, онкостатин, ингибирующий лейкемию фактор, цилиарный нейротрофический фактор, кардиотрофин-1 и кардиотрофиноподобный цитокин. Эти цитокины характеризуются общим использованием рецептора gp130 (также известного как IL-6rβ, или CD130) как сигнальной субъединицы.

В качестве миокина ИЛ-6 известен с 2000 г., и сегодня очевидно, что физическая активность и интенсивные мышечные сокращения индуцируют его синтез миоцитами скелетных мышц, а максимальный пик секреции наблюдается спустя 1–3 ч после нагрузки. Так, по данным B.K. Pedersen и M.A. Febbraio, в сыворотке человека при езде на велосипеде в течение 2 ч концентрация ИЛ-6 увеличивается в 8–11 раз, а при 3-часовой нагрузке — в 30 раз, достигая значений 25 пг/мл. Авторы отмечают, что в ходе интенсивного и длительного бега уровень ИЛ-6 может повышаться в 100 раз, что сравнимо с увеличением содержания данного цитокина при сепсисе [6]. Однако при сепсисе повышение ИЛ-6 ассоциировано с увеличением циркулирующего фактора некроза опухоли (ФНО)-α, чего не наблюдается во время ФН.

При ожирении базальный уровень ИЛ-6 повышается, так как жировая ткань является вторым по величине источником ИЛ-6 в состоянии покоя после клеток иммунной системы [84][85].

Степень повышения ИЛ-6 при ожирении коррелирует с выраженностью инсулинорезистентности в исследованиях in vivo и in vitro [86].

Величина, на которую увеличивается сывороточный уровень ИЛ-6 при физической нагрузке, определяется ее интенсивностью и продолжительностью. При физических нагрузках мышечные волокна I и II типов экспрессируют ИЛ-6, который оказывает свое действие как местно (ауто- и паракринно), так и системно. Так, на уровне скелетной мускулатуры ИЛ-6 активирует АМФ-киназу и/или фосфатидилинозитол-3-киназу через рецептор gp130rβ/IL-6Ra, что приводит к увеличению поглощения глюкозы и окислению жирных кислот, обеспечивая энергетическим субстратом сокращающиеся мышцы.

Системное действие циркулирующего ИЛ-6 реализуется преимущественно на уровне жировой ткани и печени, а также направлено на мобилизацию энергетических ресурсов организма. Так, исследования, проведенные на культурах адипоцитов человека, демонстрируют, что ИЛ-6 проявляет липолитический эффект за счет повышения активности липопротеинлипазы [87][88].

Кроме того, ИЛ-6 оказывает угнетающее влияние на действие инсулина в адипоцитах и гепатоцитах за счет подавления образования субстрата рецептора инсулина-1 (IRS-1) и трансмембранного транспортера глюкозы GLUT-4, что проявляется в уменьшении инсулинстимулированного усвоения глюкозы [89].

В гепатоцитах ИЛ-6 способствует высвобождению глюкозы, стимулирует расщепление гликогена (за счет активации гликогенфосфорилазы) и тормозит его синтез [90–92].

Молекулярный механизм угнетающего влияния ИЛ-6 на действие инсулина в печени заключается в синтезе SOSC-3 (suppressor of cytokine signaling), который ретроградно отвечает за сигнальный путь цитокина. SOSC-3 может связываться и угнетать активность как мембранного рецептора инсулина, так и IRS-1, и препятствовать проведению инсулинового сигнала [93].

Таким образом, ИЛ-6 способствует формированию инсулинорезистентности в жировой ткани и гепатоцитах при ФН для более эффективной мобилизации глюкозы и жирных кислот в качестве источников энергии.

Если в адипоцитах и гепатоцитах ИЛ-6 снижает чувствительность к инсулину, то в мышечных клетках, наоборот, усиливает его эффекты. Показано, что в присутствии ИЛ-6 улучшается действие инсулина на изолированные мышечные клетки: стимулируется усвоение глюкозы и синтез гликогена [94]. Исследования последних лет позволяют предположить, что степень повышения секреции ИЛ-6 при физической активности в первую очередь зависит от содержания гликогена в мышечных клетках: чем оно меньше, тем выше секреция цитокина [95][96].

Секреция ИЛ-6 при сократительной деятельности скелетных мышц определяется доступностью энергоносителей, а дефицит гликогена в скелетной мускулатуре стимулирует секрецию ИЛ-6. Причем эффекты ИЛ-6 на энергетический обмен могут реализовываться без участия других регуляторных систем. Так, введение ИЛ-6 в течение 3 ч здоровым добровольцам повышало липолиз, окисление жирных кислот без изменения концентрации в крови адреналина, инсулина или глюкагона в крови [97].

Таким образом, основными функциями ИЛ-6 в условиях физической активности являются мобилизация энергетических субстратов в печени и жировой ткани и обеспечение их усвоения и утилизации в скелетных мышцах.

## ОСТЕОКАЛЬЦИН

Мышечная и костная ткань тесно взаимосвязаны. Сигнальные молекулы, секретируемые костной тканью, — остеокины также оказывают системное действие на ряд органов и тканей, главным образом на мышцы. Наиболее изученным является остеокальцин (ОСК). Он представляет собой белок костного матрикса, связывающий кальций и гидроксиапатиты, синтезируется остеобластами в процессе минерализации костной ткани. Под воздействием остеокластов и при участии витамина К ОСК высвобождается в кровь. Наиболее известен как биохимический маркер костного ремоделирования.

Кроме этого, ОСК способствует пролиферации β-клеток поджелудочной железы, повышает поглощение глюкозы периферическими тканями, а также стимулирует секрецию инсулина за счет прямого действия на β-клетки и стимуляции глюкагоноподобного пептида -1 кишечника [98]. Он также увеличивается после ФН [99–101], повышает мышечную силу и способствует гипертрофии мышечных волокон. Мыши с дефицитом ОСК имеют более низкую мышечную массу [102].

Недавние исследования установили перекрестную взаимосвязь между ОСК и ИЛ-6. Так, в экспериментах на мышах показано, что после ФН отмечалось повышение концентрации обеих молекул, но при дефиците ИЛ-6 уровень ОСК не изменяется. Инъекция ИЛ-6 приводила к увеличению концентрации ОСК, что доказывает наличие перекрестной взаимосвязи между данными цитокинами [103]. Подобные исследования у людей единичны. Было показано, что при ФН увеличение ОСК зависит от секреции ИЛ-6. Применение препарата тоцилизумаба (антитела к ИЛ-6) после 12-недельного режима тренировок на выносливость приводило к подавлению продукции ОСК [103].

Таким образом, остеокальцин положительно влияет на углеводный обмен, повышая чувствительность тканей к инсулину, а также участвует в росте мышечной массы, увеличиваясь после ФН.

## ЗАКЛЮЧЕНИЕ

В последние 20 лет большое внимание уделяется изучению эндокринной функции мышечной ткани. Особые сигнальные молекулы — миокины синтезируются миоцитами и высвобождаются в кровоток в ответ на сокращение мышечных волокон, взаимодействия с другими органами, в первую очередь жировой тканью, печенью и головным мозгом. Наиболее изученными на сегодняшний день являются миостатин, ирисин, ИЛ-6, декорин, FGF-21. Миокины играют роль в реализации многочисленных процессов, таких как миогенез, остеогенез, термогенез, липолиз, повышение чувствительности тканей к глюкозе. Изучение миокинов поможет ответить на важные вопросы, ведущие к пониманию механизмов, лежащих в основе ожирения и метаболических осложнений, а также последствий малоподвижного образа жизни. В перспективе сигнальные молекулы мышечной ткани могут стать терапевтическими мишенями при данных состояниях. Учитывая, что миокины стимулируются сокращением мышц, их изучение раскрывает механизмы реализации положительных эффектов физической активности.
